# Weak regulations threaten the safety of consumers from harmful weight-loss supplements globally: results from a pilot global policy scan

**DOI:** 10.1017/S1368980023000708

**Published:** 2023-09

**Authors:** Funmbi T Okoya, Monique Santoso, Amanda Raffoul, Maya Azar Atallah, S Bryn Austin

**Affiliations:** 1 Department of Epidemiology, Harvard T.H. Chan School of Public Health, Boston, MA, USA; 2 Division of Adolescent/Young Adult Medicine, Boston Children’s Hospital, Boston, MA 02115, USA; 3 Department of Pediatrics, Harvard Medical School, Boston, MA, USA; 4 Department of Rehabilitation & Sport Sciences, Faculty of Health & Social Sciences, Bournemouth University, Bournemouth, England; 5 Department of Social and Behavioral Sciences, Harvard T.H. Chan School of Public Health, Boston, MA, USA

**Keywords:** Dietary supplements, weight loss, advertising, regulation

## Abstract

**Objective:**

To pilot a global policy scan assessing how governments worldwide regulate weight-loss supplements (WLS).

**Design:**

Experts on WLS policies from thirty countries that varied by World Bank income classification, with five from each of the six WHO regions, completed an online survey on WLS regulation in their country. The survey covered six domains: legal frameworks; pre-market requirements; claims, labelling, and advertisements; product availability; adverse events reporting; and monitoring and enforcement. Percentages were calculated for presence or absence of a type of regulation.

**Setting:**

Experts were recruited through websites of regulatory bodies and professional LinkedIn networks and scientific article searches on Google Scholar.

**Participants:**

Thirty experts, one from each country (i.e. researchers, regulators, other experts in food and drug regulation).

**Results:**

WLS regulations varied widely across countries, and a number of gaps were identified. One country (Nigeria) has a minimum legal age to purchase WLS. Thirteen countries reported independently evaluating the safety of a new WLS product sample. Two countries have limitations on where WLS can be sold. In eleven countries, reports on adverse events related to WLS are publicly available. In eighteen countries, safety of new WLS is to be established through scientific criteria. Penalties for WLS non-compliance with pre-market regulations exist in twelve countries and labelling requirements in sixteen countries.

**Conclusions:**

Results of this pilot study document wide variability in national WLS regulations globally, exposing many gaps in important components of consumer protection regulatory frameworks for WLS, which likely put consumer health at risk.

## Introduction

International food and drug regulators from countries including the EU, USA, China, Japan, India, Canada and Australia estimated that over 80 % of the world’s population uses dietary supplements or herbal medicine in the 2018 Global Summit on Regulatory Science, established under the leadership of the US Food and Drug Administration (FDA)^(1)^. Thakkar et al. define dietary supplements as ‘supplements for health benefits with no or limited claim on therapeutic effects’, yet there is a lack of global consensus on how these products are defined^(1)^, which has hindered progress in research on the fast-growing marketplace for dietary supplements^(2,3)^. The lack of consensus on the definition also gives rise to challenges around regulatory frameworks to ensure efficacy and safety of dietary supplements for consumers^(2)^. Prior research has observed that even among countries that are similar culturally, legally and economically, policies relating to the regulation of dietary supplements vary markedly^(2)^.

In particular, regulations pertaining to weight-loss supplements (WLS) are lacking globally^(4)^, and challenges to regulation include a lack of means for assuring the quality, safety and efficacy of WLS; the extent to which quality testing is required; and monitoring to detect potential adverse events^(4)^. Weak regulation creates space for the growing dietary supplements industry, currently valued at USD $300 billion globally, to sell ineffective, dangerous and potentially fatal WLS products to consumers^(5)^. Among these consumers are adolescents, who may be especially susceptible to use of WLS due to body image concerns that can worsen during this developmental period^(6,7)^. In addition to adolescents, target groups for WLS include military personnel, weight class sports, young adults and women^(8–11)^.

A growing body of research has demonstrated that WLS are often adulterated with illegal and toxic ingredients^(12–14)^, which have been associated with haemorrhagic strokes, hepatotoxicity and even sudden death^(15–18)^. Even when they do not cause liver failure and death, they can have serious negative side effects including anxiety, insomnia and eating disorders, leading to emergency room visitations^(19–23)^. For instance, in 2019, a 23-year-old woman was diagnosed with acute liver failure that was associated with a WLS she had been taking for several months^(24)^. Additionally, in 2015, a 21-year-old woman died after taking a WLS that she bought online which was later found to be adulterated with DNP, a highly toxic industrial chemical^(25)^.

Moreover, there are very few studies showing the safety and efficacy of WLS, which are widely available and marketed to consumers^(26–29)^. Within the EU, a systematic review found that 63 % of WLS contained unapproved ingredients^(30)^. Within a random sample of 137 WLS used in the United Arab Emirates, 18 % were found to have sizable concentration of undeclared pharmaceuticals^(31)^. In the USA, 40·9 % of WLS have been found to be adulterated with more than one unapproved ingredient^(32)^. The most common adulterant in WLS is sibutramine, a drug withdrawn from the market in 2010 due to cardiovascular risks, which was found present in 84·9 % of adulterated WLS in the USA^(32)^. In a 2012 survey of 3500 US adults^(26)^, a majority of users (65 %), compared with non-users (40 %), falsely believed WLS were safe, with over half of users incorrectly believing that they have been approved for safety by a government agency, such as the US FDA, prior to sale. Research within the USA has indicated that girls and women are two times more likely to use WLS in their lifetimes than boys and men^(33)^. Results from the US National Health and Nutrition Examination Survey found that among adults who have attempted to lose weight, African American and Hispanic were more likely than White adults, individuals with a high school degree or less were more likely than those with a higher degree, and lower income households were more likely than higher income households to use WLS^(11)^. Moreover, recent findings reveal higher financial precarity and psychological distress, and food insecurity was associated with greater risk of WLS use during the first year of the COVID-19 pandemic^(34)^.

With the rapid market growth in WLS and the threat they pose to public health, it is important to monitor regulation across nations with the goal of identifying best practices and gaps where regulations could be strengthened to protect consumers. Global policy scans have been used to monitor rapidly expanding consumer products, such as e-cigarettes^(35,36)^, that pose a threat to public health worldwide. While the WHO’s Nutrition and Food Safety Office tracks worldwide nutrition-related regulation, its Global Nutrition Policy Review^(37)^ does not include dietary supplements nor WLS and does not document how different countries regulate these products.

The aim of this pilot study was to identify how national governments regulate dietary supplements sold for weight loss globally. We hypothesised that, despite the health risks associated with these products, WLS are widely underregulated globally with few if any limitations on their availability and regulators do not adequately assess WLS safety, efficacy or claims before WLS enter the market.

## Methods

### Survey design

We developed a survey covering six domains of WLS regulation in consultation with a key informant with extensive national consumer product regulation expertise. The domains were as follows: (1) legal frameworks (e.g. ‘Does the legislation in your country have a specific legal framework (laws or regulations) for WLS?’); (2) pre-market requirements (e.g. ‘How does the regulator(s) in your country determine the efficacy of a new WLS?’); (3) claims, labelling and advertisements (e.g. ‘Are there specific labelling requirements for WLS (e.g. warnings, disclaimers, instructions for consumption, etc.) in your country?’); (4) product availability (e.g. ‘Are there limitations on where WLS can be sold in your country?’); (5) adverse events (e.g. ‘Are the reports on adverse events related to WLS available to the public in your country?’); and (6) monitoring and enforcement (e.g. ‘Which of the following does the regulator(s) in your country monitor regarding WLS?’). The full survey can be accessed at https://www.hsph.harvard.edu/striped/global-policy-scan/.

The survey was prepared in English with a French translation available upon request and hosted on an online Qualtrics survey platform. Items were presented in multiple-choice format, followed by open-text prompts for participants to provide additional information. All questions had a ‘don’t know’ response option in case participants did not have the information necessary to answer a question and an ‘other’ response. We defined WLS as any dietary supplement promoted for weight loss. Claims were defined as statements made about a product’s content or effects (i.e. health, nutritional or functional). An adverse event was defined as any undesirable experience associated with the use of a product by a patient or consumer.

### Sampling and data collection

We used a non-random purposive sampling strategy to select five countries with varying World Bank country-level income status from each of the six WHO regions. We then sought one expert on drug and dietary supplements regulation from each of those countries to complete our survey. We made multiple attempts to recruit appropriate regulators employed by their national government. In countries where either the government regulator declined to participate or where, after multiple outreach attempts, we did not receive a response from the government regulator, we then identified an in-country expert. Experts were identified from websites of respective regulatory bodies, research papers on Google Scholar and the authors’ professional LinkedIn networks.

An initial invitation email was sent to each identified expert, containing an introduction to the study purpose with the survey link included, to a single expert in each of the selected thirty countries. Follow-up emails were sent every 2 weeks and 4 weeks after initial contact, or until survey commencement. Of all experts contacted, eighteen did not respond after 2 months, did not commence the survey after follow-up emails, and/or mentioned that they did not intend to take part in the study. If experts did not respond after 2 months, did not commence the survey after the follow-up emails or responded that they did not intend to take part in the survey, additional contacts were made via email, phone and LinkedIn to identify an appropriate alternate expert from that country to fill out the survey.

Similarly, seventeen experts provided either incomplete responses with less than 75 % progress or had at least a month of inactivity despite follow-up reminders, resulting in our termination of their participation. In total, 129 experts were contacted, and we received thirty-two completed surveys. For two countries, inconsistencies in response times and rates resulted in two expert responses being obtained. When this occurred, the team prioritised survey responses from individuals working in regulatory agencies.

Data collection ended when complete survey responses from one expert in each of the thirty countries were obtained. Data collection occurred from July 2021 to November 2021. As this pilot study gathered data solely on national regulations, institutional review board approval was not required.

### Analysis

Descriptive statistics were obtained from Qualtrics, and percentages were calculated for presence or absence of a type of regulation. ‘Don’t know’ responses were excluded from calculations of percentages but listed in the tables. Qualitative responses that fell under the ‘Other’ category within multiple-choice questions were reviewed by two coders (FO and MS) and identified as to whether they could be recategorised as ‘Yes’, ‘No’ or ‘Don’t Know’, or whether the response presented new information that needed to be considered. All conflicts were discussed between coders.

## Results

Sample characteristics are summarised in Table 1. Experts were categorised based on professional credentials with most identifying as regulators/administrators (30 %), researchers (26·7 %) and pharmacists (20 %) working in the food and drug space. Five countries participated from each of the six WHO regions: African region (Uganda, United Republic of Tanzania, Zimbabwe, Botswana and Nigeria), Region of the Americas (Venezuela, Canada, Brazil, Ecuador and USA), Eastern Mediterranean Region (Lebanon, Morocco, Islamic Republic of Iran, Pakistan and Saudi Arabia), European Region (Belgium, United Kingdom of Great Britain and Northern Ireland, Ukraine, Latvia, Turkey), South-East Asian Region (Sri Lanka, India, Bangladesh, Thailand and Indonesia) and Western Pacific Region (Australia, Malaysia, China, Singapore and Philippines).

**Table 1:** Characteristics of thirty expert respondents from thirty countries participating in a global policy scan pilot study assessing national regulation of weight-loss supplements

Characteristics of expert respondents and countries	*n*	%
Profession (check all that apply)		
Regulator/administrator	9	30
Pharmacist	6	20
Other healthcare professional	4	13·3
Public health professional	4	13·3
Researcher	8	26·7
Other professionals and researchers with food and drug expertise	6	20
WHO region		
The African region	5	16·7
The Region of the Americas	5	16·7
The Eastern Mediterranean region	5	16·7
The European region	5	16·7
The South-East Asian region	5	16·7
The Western Pacific region	5	16·7
World Bank Country Income Classification		
Low income	1	3·3
Middle income	20	66·7
High income	9	30·0

### Domains (1) Legal framework and (2) Pre-market regulation of WLS

Of the thirty countries sampled, experts surveyed from seven countries (Canada, Belgium, Morocco, Pakistan, Indonesia, Nigeria and Saudi Arabia) reported that a specific legal framework for WLS exists, and of those that do not have a specific legal framework for WLS, countries variably regulated WLS as drug only (10 %), as drug and food or drug and supplements (35 %), as food only (25 %), or as supplements only (20 %). Of the countries sampled, 40 % of surveyed experts stated that there are no limits to the ingredients or substances permitted in WLS. Less than two-thirds (64 %) of the experts in countries surveyed indicated a requirement for the safety of a new WLS to be established through scientific criteria, 63 % have a registration requirement for WLS and 44 % have a licensing requirement for WLS (Table 2).

**Table 2:** Expert responses describing national legal frameworks and pre-market regulation of weight-loss supplements (WLS) across thirty countries (*n* 30)

Questions†	*n* of yes responses (excluding ‘Don’t Know’)	%	Number of ‘Don’t know’ responses	%
Country has a specific legal framework for WLS (*n* 23)	7	29·2	6	20·0
Country limits the ingredients or substances used in WLS (*n* 25)	15	60·0	5	16·7
How the safety of a new WLS is determined* (*n* 25)	–		5	16·7
Independent evaluation of product samples	13	52·0	–	
Safety information provided by the manufacturer	19	76·0	–	
Literature search by regulator(s)	2	8·0	–	
Regulator(s) does not confirm safety	1	4·0	–	
Country requires the safety of a new WLS to be established through scientific criteria (*n* 28)	18	64·3	2	6·7
How the efficacy of a new WLS is determined* (*n* 26)	–		4	13·3
Independent evaluation of product samples	9	34·6	–	
Efficacy information provided by manufacturer	11	42·3	–	
Literature search by regulator(s)	3	11·5	–	
Regulator(s) does not confirm efficacy	12	46·2	–	
Country requires the efficacy of a new WLS to be established through scientific criteria (*n* 25)	13	52·0	5	16·7
New imported WLS are subject to the same regulations as new locally produced WLS (*n* 26)	22	84·6	4	13·3
Country has a registration requirement for WLS (*n* 30)	19	63·3	0	0·0
Country has a licensing requirement for WLS (*n* 23)	10	43·5	7	23·3
Country has a registration requirement for WLS manufacturers/producers (*n* 25)	19	76·0	5	16·7
Country has a licensing requirement for WLS manufacturers/producers (*n* 23)	14	60·9	7	23·3

* Question allowed respondents to ‘check all that apply’; responses are not mutually exclusive.

† Sample size varies depending on number of ‘don’t know’ responses. Sample sizes listed per question have excluded the number of ‘don’t know’ responses for that question.

### Domains (3) Claims, labelling, advertisements and (4) Product availability

Table 3 summarises responses to items surrounding the regulation of claims, labelling, advertisements and availability of WLS. Nearly three-quarters (74 %) of experts in the countries surveyed reported certain claims related to WLS are prohibited, and 52 % stated requirements that claims have a scientific basis. Surveyed experts stated that specific labelling requirements for WLS such as warnings, disclaimers and instructions for consumption are required in 61 % of the countries surveyed. Over half of the experts in the countries surveyed reported the presence of regulatory limitations on WLS advertising on TV and cinema, billboards and banners, radio, printed media, and the internet. Of the countries with limitations on WLS advertising, 50 % of experts stated their countries have specific limitations on advertising WLS to children. Only the expert from Nigeria reported the presence of a minimum legal age (18 years) to purchase WLS. Twenty-four per cent of experts in countries within the sample reported that their countries have specific laws and regulations regarding the online sale and purchase of WLS.

**Table 3:** Expert responses on claims, labelling, advertisements, availability, adverse events, and monitoring and enforcement of weight-loss supplements (WLS) across thirty countries (*n* 30)

Questions†	*n* of yes responses (excluding ‘Don’t Know’)	%	Number of ‘Don’t know’ responses	%
How claims related to WLS are regulated* (*n* 27)	–		3	10·0
Certain claims are prohibited	20	74·1	–	
Pre-approval of claims is required	9	33·3	–	
Claims are required to have a scientific basis	14	51·9	–	
Claims are not regulated	5	18·5	–	
Country has specific labelling requirements for WLS (e.g. warnings, disclaimers, instructions for consumption, etc.) (*n* 28)	17	60·7	2	6·7
Country has regulatory limitations on advertising WLS (*n* 25)	14	56·0	5	16·6
Specific limitations on advertising WLS to children (*n* 12)‡	6	50·0	9	30·0
Require disclaimers or warnings to be included in WLS advertisements (*n* 22)	11	50·0	8	26·7
Have limitations on where WLS can be sold (*n* 27)	2	7·4	3	10·0
Require certain WLS to be sold only with a prescription (*n* 27)	7	25·9	3	10·0
Have a minimum legal age to buy WLS (*n* 24)	1	4·2	6	20·0
Have specific laws and regulations regarding the online sale and purchase of WLS (*n* 25)	6	24·0	5	16·7
Require the manufacturer, importer or another agent (e.g. pharmacy) to report adverse events related to the WLS experienced by consumers (*n* 27)	15	55·6	3	10·0
Have an official process for reporting adverse events experienced after consuming a WLS (*n* 27)	22	81·5	3	10·0
Make reports on adverse events related to WLS available to the public (*n* 27)	11	44·0	3	10·0
Take steps to determine whether a recall is warranted when adverse event related to WLS is reported (*n* 29)	22	75·9	1	3·3
Uses penalties or other sanctions for non-compliance with:* (*n* 19)			11	36·7
Pre-market regulation	12	63·2	–	
Claims	15	78·9	–	
Labelling	16	84·2	–	
Advertisements	12	63·2	–	
Availability	4	21·1	–	
Adverse event reporting	10	52·6	–	
No sanctions	1	5·3	–	

* Question allowed respondents to ‘check all that apply’; responses are not mutually exclusive.

† Sample size varies depending on number of ‘don’t know’ responses. Sample sizes listed per question have excluded the number of ‘don’t know’ responses for that question.

‡ Question administered only to twelve respondents who answered ‘Yes’ to ‘Country has regulatory limitations on advertising WLS’.

### Domains (5) Adverse events and (6) Monitoring and enforcement of WLS

Responses to items surrounding adverse events and monitoring and enforcement of WLS are summarised in Table 3. Fifty-six per cent of experts reported that their countries require the manufacturer, importer or another agent to report adverse events related to the WLS use experienced by consumers, 44 % of experts stated that their countries require reports on adverse events related to WLS be made available to the public, and 76 % of experts mentioned that their countries take steps to determine whether a recall is warranted when adverse event related to WLS is reported. Expert survey responses also illustrated that countries may impose penalties or other sanctions for non-compliance with pre-market regulation (63 %), claims (79 %), labelling (84 %), advertisements (63 %), availability (21 %) and adverse event reporting (53 %).

## Discussion

This pilot study provides an initial snapshot of experts’ understanding of WLS regulation in thirty countries. As hypothesised, our findings indicate that WLS are under-regulated globally, which is especially concerning given the unprecedented growth in dietary supplement sales during the COVID-19 pandemic in stores and online^(3,38,39)^. While we assessed regulations in only thirty of the world’s 195 countries, this study’s survey and findings will provide a rigorous, comparative tool for systematic assessment of national regulations on WLS that is well suited for future investigations of WLS regulations globally.

Few countries have regulations on advertising WLS to children, and only the expert in Nigeria reported a minimum legal age to purchase WLS. Experts in most surveyed countries indicated that a protocol exists for recall of a supplement when adverse events related to WLS are reported (82 %); yet only in a few countries were these reports made available to the public (44 %) or required manufacturers, importers, and other related agents to report adverse events (56 %). As a result, it is not clear how often government regulators or the public are made aware when adverse events occur, allowing misinformed positive consumer perception of these products to persist^(40)^. Furthermore, our findings indicate that few nations have penalties and sanctions within their legal framework relating to non-compliance with pre-market regulation, claims, labelling, advertising, availability and adverse event reporting of WLS, which poses risks to consumer safety, as companies can continue their practices unfettered by government sanction for non-compliance.

Our findings are consistent with one other study on the regulation of WLS in different countries, which found a lack of regulations relating to dietary supplements generally, and, when regulations did exist, they often were not strictly enforced^(2)^. Various studies worldwide have found that WLS can contain adulterated ingredients that can lead to deadly effects for consumers^(12,13,18)^. Implementation of stronger regulations on WLS at regional, such as those implemented within the European Union, and national levels will be essential to protect consumers, especially children.

This pilot study is limited in generalisability given that our findings are based on a non-random, purposive sample that included only thirty countries, of which only Uganda was a low-income country according to World Bank classifications. In this vein, we also note that the sample size of experts was limited and that not all experts were individuals working for their nations’ regulatory agencies, with some identifying as pharmacists, public health professionals, and researchers in the food and drug space. We also recognise that regulatory terms vary across countries and regions, particularly with different languages, potentially leading to heterogeneity in interpretation of our survey questions. For example, one question addressing the licensing requirement for WLS may have been interpreted to address both licensing and registration, as one expert mentioned that the terms were used interchangeably in their country. Importantly, we were not able to collect data to assess how often penalties are imposed when violations occur nor to assess how robust enforcement efforts are in each country. Though our findings provide new insight into the extant WLS regulatory frameworks in each of the sampled countries, this study does not provide information on enforcement as it was outside the scope of this pilot study.

Another limitation is the difficulty of defining WLS, given the lack of expert consensus and varying definitions by national jurisdictions. Our study therefore instructed respondents to broadly define WLS as ‘any dietary supplement promoted for weight loss’. We also note that product names and brands vary across countries and, therefore, we did not list or refer to any country-specific brand names. Moreover, this study surveyed expert opinions on WLS regulation in their respective nations, and we did not conduct legal analysis of the text of current law and policy in these nations to support the claims of experts. Future research should include legal analysis of the text of regulatory law and policy in each country, rather than relying on expert knowledge, which could more accurately identify differences in the definitions of safety, quality and efficacy by country. Should legal analysis not indicate these differences adequately (e.g. some countries do not provide standards for efficacy), then a follow-up survey could be sent to experts to further elicit their insights into these definition differences between countries.

Similarly, given that this pilot study included only one expert for every surveyed country, future research would benefit from surveying multiple experts in these countries and analysing the similarities and differences in their responses. Ideally, the experts surveyed would be professionals working in national or international regulatory bodies to provide the most accurate and timely information on national regulations on WLS. Future research should include more low- and low-middle income countries to better illuminate differences in WLS regulation by country income classification.

The WHO Nutrition and Food Safety programme provides global leadership by documenting nutrition and food safety regulations worldwide in its Global Nutrition Policy Review^(29)^. We strongly recommend that WHO similarly begin to track regulations worldwide on dietary supplements, especially WLS given their well-documented risks to consumers. We recommend rigorous pre-screening for safety of WLS before they are sold in markets, the enforcement of prohibition of unsubstantiated health claims and age restrictions for purchase of WLS to promote consumer safety^(4,41)^. Our pilot study methodology can serve as a model for WHO to begin this vitally needed tracking of regulation globally and would allow for systematic and rigorous national policy assessments to help to ensure consumers worldwide are protected from the harms of WLS.

## Conclusion

Regulatory frameworks surrounding the sale, advertising and enforcement of WLS globally are largely insufficient, particularly around regulations designed to protect children. Given the robust literature illustrating the deleterious effects that WLS can have on consumer safety and children’s physical and mental health, it is crucial for national governments to develop regulations for WLS to impose restrictions on their ingredients, claims and advertisements, and availability.
